# Circulating matrix metalloproteinases and tissue metalloproteinase inhibitors in patients with idiopathic pulmonary fibrosis in the multicenter IPF-PRO Registry cohort

**DOI:** 10.1186/s12890-020-1103-4

**Published:** 2020-03-14

**Authors:** Jamie L. Todd, Richard Vinisko, Yi Liu, Megan L. Neely, Robert Overton, Kevin R. Flaherty, Imre Noth, L. Kristin Newby, Joseph A. Lasky, Mitchell A. Olman, Christian Hesslinger, Thomas B. Leonard, Scott M. Palmer, John A. Belperio, on behalf of the IPF-PRO Registry investigators, Wael Asi, Albert Baker, Scott Beegle, Rany Condos, Francis Cordova, Daniel A. Culver, Tracey Luckhardt, Daniel Dilling, Marilyn Glassberg, Mridu Gulati, Kalpalatha Guntupalli, Nishant Gupta, David Hotchkin, Tristan Huie, Robert Kaner, Hyun Kim, Maryl Kreider, Lisa Lancaster, David Lederer, Doug Lee, Timothy Liesching, Randolph Lipchik, Jason Lobo, Yolanda Mageto, Prema Menon, Lake Morrison, Andrew Namen, Justin Oldham, Rishi Raj, Murali Ramaswamy, Tonya Russell, Paul Sachs, Zeenat Safdar, Barry Sigal, Leann Silhan, Mary Strek, Sally Suliman, Jeremy Tabak, Rajat Walia, Timothy P. Whelan

**Affiliations:** 1 Duke Clinical Research Institute, Durham, NC USA; 2 Division of Pulmonary, Allergy, and Critical Care Medicine, Department of Internal Medicine, Duke University Medical Center, DUMC Box 103002, Durham, NC 27710 USA; 3 Boehringer Ingelheim Pharmaceuticals Inc., Ridgefield, CT USA; 4 Division of Pulmonary and Critical Care Medicine, University of Michigan, Ann Arbor, MI USA; 5 University of Virginia, Charlottesville, VA USA; 6 Duke Clinical & Translational Science Institute, Durham, NC USA; 7 School of Medicine, Tulane University, New Orleans, LA USA; 8 Department of Inflammation and Immunity and Respiratory Institute, Cleveland Clinic, Cleveland, OH USA; 9 Boehringer Ingelheim Pharma GmbH & Co. KG, Biberach, Germany; 10 David Geffen School of Medicine at UCLA, Los Angeles, CA USA

**Keywords:** Extracellular matrix, Biomarkers, Fibrosis, Interstitial lung diseases, Observational study

## Abstract

**Background:**

Matrix metalloproteinases (MMPs) and tissue inhibitors of MMPs (TIMPs) play important roles in the turnover of extracellular matrix and in the pathogenesis of idiopathic pulmonary fibrosis (IPF). This study aimed to determine the utility of circulating MMPs and TIMPs in distinguishing patients with IPF from controls and to explore associations between MMPs/TIMPs and measures of disease severity in patients with IPF.

**Methods:**

The IPF cohort (*n* = 300) came from the IPF-PRO Registry, an observational multicenter registry of patients with IPF that was diagnosed or confirmed at the enrolling center in the past 6 months. Controls (*n* = 100) without known lung disease came from a population-based registry. Generalized linear models were used to compare circulating concentrations of MMPs 1, 2, 3, 7, 8, 9, 12, and 13 and TIMPs 1, 2, and 4 between patients with IPF and controls, and to investigate associations between circulating levels of these proteins and measures of IPF severity. Multivariable models were fit to identify the MMP/TIMPs that best distinguished patients with IPF from controls.

**Results:**

All the MMP/TIMPs analyzed were present at significantly higher levels in patients with IPF compared with controls except for TIMP2. Multivariable analyses selected MMP8, MMP9 and TIMP1 as top candidates for distinguishing patients with IPF from controls. Higher concentrations of MMP7, MMP12, MMP13 and TIMP4 were significantly associated with lower diffusion capacity of the lung for carbon monoxide (DL_CO_) % predicted and higher composite physiologic index (worse disease). MMP9 was associated with the composite physiologic index. No MMP/TIMPs were associated with forced vital capacity % predicted.

**Conclusions:**

Circulating MMPs and TIMPs were broadly elevated among patients with IPF. Select MMP/TIMPs strongly associated with measures of disease severity. Our results identify potential MMP/TIMP targets for further development as disease-related biomarkers.

## Background

Idiopathic pulmonary fibrosis (IPF) is a progressive interstitial lung disease associated with high mortality [1]. Two anti-fibrotic agents, nintedanib and pirfenidone, have been approved for the treatment of IPF and demonstrated to slow the progression of the disease [2, 3]. However, the diagnosis and management of IPF remain challenging, with no clinically available biomarkers to serve as adjuncts in diagnosis or prediction of prognosis or treatment response.

The pathobiology of IPF involves excess production of extracellular matrix (ECM) and dysregulated matrix remodeling [4]. Matrix metalloproteinases (MMPs) are a family of zinc-dependent endopeptidases important in ECM degradation. Expression of MMPs and their physiological inhibitors, tissue inhibitors of MMPs (TIMPs), is tightly regulated in the lung, with notable upregulation during lung development, tissue injury, and host defense [5].

Several basic and clinical studies have underscored the importance of MMPs and TIMPs in the pathobiology of IPF, as recently reviewed [6]. In particular, murine models of bleomycin-induced pulmonary fibrosis demonstrated increased expression of MMPs and TIMPs, while mice with genetic deletions in select MMPs had reduced lung fibrosis after bleomycin administration compared with wild type mice [6, 7]. Patients with IPF showed increased MMP and TIMP expression in the lungs [8–10], including in structural cells (for example, the epithelium) and immune cells (for example, interstitial macrophages) [8]. Circulating levels of MMPs 1, 3, 7, 8, and 9 have been shown to be elevated in patients with IPF [9, 11] and higher circulating levels of MMP7 to be associated with more severe disease [11], a higher risk of disease worsening over a 3-year period [12], and shorter survival time [13]. However, there remains a relative paucity of information on the full range of MMPs and TIMPs detectable in the blood of patients with IPF and their utility as biomarkers. We sought to determine expression of MMPs 1, 2, 3, 7, 8, 9, 12, and 13 and TIMPs 1, 2, and 4 in a cohort of well-characterized patients with IPF, to understand if combinations of MMPs and TIMPs could distinguish patients with IPF from controls, and to investigate associations between MMPs/TIMPs and measures of IPF severity.

## Methods

### IPF cohort

The IPF cohort was drawn from the multicenter observational US Idiopathic Pulmonary Fibrosis Prospective Outcomes (IPF-PRO) Registry (NCT01915511) [14] that enrolled patients with IPF that was diagnosed or confirmed at the enrolling center in the past 6 months. The cohort for this analysis consisted of 300 patients enrolled by 1 February 2017, who had an enrollment blood sample, data on critical clinical variables at enrollment, and an assessment form indicating the certainty of the IPF diagnosis (definite, probable, possible) determined by the investigator according to the 2011 American Thoracic Society/European Respiratory Society/Japanese Respiratory Society/Latin American Thoracic Association (ATS/ERS/JRS/ALAT) diagnostic guidelines [15].

### Control cohort

The control cohort was drawn from the Measurement to Understand the Reclassification of Disease of Cabarrus/Kannapolis (MURDOCK) Study, a registry of adult residents of North Carolina in which self-reported health information and biological samples are collected [16]. To ensure that the control cohort had a similar age, race and ethnicity distribution to the IPF cohort, participants considered for inclusion as controls were White, non-Hispanic and aged 60 to 80 years. Participants were excluded if they had self-reported respiratory disease, cancer, or autoimmune disease at enrollment or during follow-up, were active smokers, had active second-hand tobacco exposure, or reported use of respiratory-targeted medications or immunomodulators. Random sampling with stratification by sex and smoking status (ever versus never) was used to select 100 controls with a similar distribution of these characteristics to the IPF cohort.

### MMP and TIMP quantification

Enrollment plasma samples were assayed for antigenic levels of MMPs 1, 2, 3, 7, 8, 9, 12, and 13 and TIMPs 1, 2, and 4 (ThermoFisher; Vienna, Austria). MMPs 2, 3, 9 and TIMP1 and MMPs 1, 7, 8, 12, and 13 were quantified using multiplexed luminex immunoassays. TIMP2 and TIMP4 were quantified by ELISA. Samples that fell below the standard curve for MMP1 (*n* = 23 IPF and *n* = 17 control), MMP8 (*n* = 48 IPF and *n* = 63 control), MMP12 (*n* = 7 IPF and *n* = 4 control), and MMP13 (*n* = 10 IPF and *n* = 3 control) were extrapolated if feasible or assigned a concentration of half the minimum observed value. No samples fell below the standard curve for MMPs 2, 3, 7, or 9 or TIMPs 1, 2, and 4.

### Statistical analyses

All statistical analyses were completed in SAS version 9.4 or R version 3.5.1. Generalized linear modeling was used to compare MMP and TIMP concentrations between patients with IPF and controls. The data were log10 transformed to more closely meet the distribution assumptions for linear models. Descriptive box-plots were generated from the log10 transformed data. The results of the statistical analyses were back-transformed to the original scale and described as geometric means and geometric mean ratios of the IPF versus control groups. Correction for multiple comparisons was performed using the Benjamini-Hochberg method to control the false discovery rate at 5%.

Multivariable analyses were performed to assess whether a set of MMPs or TIMPs could differentiate patients with IPF from controls, and log10 data were centered, scaled, and Box-Cox transformed to improve model efficiency. No MMPs or TIMPs were highly correlated (Pearson correlation coefficient of ≥0.9) so all analytes were retained in the analysis. The data from the 400-patient cohort were randomly divided into training (*n* = 300; 75%) and test sets (*n* = 100; 25%) using stratified sampling to retain the 3:1 ratio in each set. Three linear models (penalized logistic regression, linear discriminant analysis, partial least squares) and 4 non-linear models (K-nearest neighbors, support vector machines, recursive partitioning [single tree], random forests [boosted trees]) were fit. Covariates for age and sex were included in each model. When fitting each model, 10-fold cross validation was used to choose the optimal tuning parameter based on the area under the receiver operating curve. Operating characteristics (accuracy, sensitivity, specificity, area under the receiver operating curve) were determined in the training set and then evaluated in the test set. Variable importance measures were determined for the best performing models [17]. The model with the best performance based on the area under the curve (AUC) was also refit using the full 400-patient cohort.

For univariate analyses in the IPF cohort, linear regression models were employed on the log10 transformed data to determine the association between circulating levels of each MMP/TIMP and three measures of disease severity, analyzed as continuous variables: FVC % predicted, DL_CO_ % predicted, and the composite physiologic index (CPI), which correlates with the extent of fibrosis on radiography in patients with IPF [18]. The National Health and Nutrition Examination Survey (NHANES) III reference eqs [19]. were used to calculate the % predicted values for FVC and FEV1, forced expiratory volume in 1 s (FEV_1_), and the reference equations developed by Crapo and Morris [20] were used to calculate the % predicted values for DLco. The linear regression analyses were repeated adjusting for treatment (nintedanib, pirfenidone, neither) at enrollment. The Benjamini-Hochberg method was used to control the false discovery rate at 5%. For each disease severity measure, the estimated coefficients and confidence intervals from the linear regression model were used to calculate the estimated difference in the disease severity measure between the median MMP/TIMP concentration in patients in the lowest tertile of disease severity and the median concentration in patients in the highest tertile of disease severity.

In multivariable analyses of the IPF cohort, FVC % predicted, DL_CO_ % predicted, and CPI were modeled separately to examine their relationship with sets of MMPs/TIMPs. Pairwise correlation analysis indicated that no MMPs or TIMPs were highly correlated (Pearson correlation coefficient of ≥0.9) so all were retained in the analysis. The performance of 2 linear models (partial least squares and penalized linear regression) and 4 non-linear models (K-nearest neighbors, support vector machines, recursive partitioning [single tree], random forests [boosted trees]) was measured on training (*n* = 225; 75%) and test (*n* = 75; 25%) sets. Covariates for age, sex, current use of nintedanib, and current use of pirfenidone were included in each model. While fitting each model, 10-fold cross-validation was used to choose the optimal tuning parameter in the training set, minimizing the root mean squared error (RMSE). R-squared was computed.

## Results

### Cohort characteristics

In the control cohort (*n* = 100), the median (Q1, Q3) age was 66.0 (63.0, 71.5) years, 74% were men, 68% were former smokers (Table 1). In the IPF cohort (*n* = 300), the median (Q1, Q3) age was 70.0 (65.0, 75.0) years, 74% were men, 94% were white and 67% were former smokers (Table 1). Most patients with IPF (73%) were characterized as having definite IPF; 10.3% were determined by the enrolling physician to have clinically significant emphysema on CT scan and 54% were taking nintedanib or pirfenidone. Median (Q1, Q3) FVC % predicted was 69.7 (61.0, 80.2), DL_CO_ % predicted was 40.6 (31.7, 49.4) and CPI was 53.5 (46.6, 60.5). The lower and upper tertile cutpoints for these measures are shown in Additional file 1.

**Table 1:** Characteristics of the IPF and control cohorts

Characteristic	IPF (*N* = 300)	Control (*N* = 100)
Age (years)	70.0 (65.0, 75.0)	66.0 (63.0, 71.5)
Male	223 (74.3%)	74 (74%)
Race		
White	281 (93.7%)	100 (100%)
Black or African-American	8 (2.7%)	0 (0%)
Asian	6 (2.0%)	0 (0%)
Other	5 (1.7%)	0 (0%)
Ethnicity: Hispanic or Latino	8 (2.7%)	0 (0%)
Smoking		
Past	202 (67.3%)	68 (68%)
Never	96 (32.0%)	32 (32%)
Current	2 (0.7%)	0 (0%)
Diagnostic criteria^a^		
Definite IPF	220 (73.3%)	–
Probable IPF	63 (21.0%)	–
Possible IPF	17 (5.7%)	–
Presence of emphysema on CT	31 (10.3%)	–
Supplemental oxygen use at rest	59 (20.0%)^b^	–
Pulmonary function measures		
FEV_1_ (L)	2.2 (1.8, 2.7)	–
FEV_1_ (% predicted)	77.4 (68.0, 89.1)	–
FVC (L)	2.7 (2.2, 3.2)	–
FVC (% predicted)	69.7 (61.0, 80.2)	–
FEV_1_/FVC ratio	74.1 (72.8, 89.6)	–
DL_CO_ (mL/min/kPa)	12.0 (8.6, 14.7)	–
DL_CO_ (% predicted)	40.6 (31.7, 49.4)	–
CPI	53.5 (46.6, 60.5)	–
Antifibrotic drug use		
Nintedanib	56 (18.7%)	0 (0%)
Pirfenidone	106 (35.3%)	0 (0%)
Neither	138 (46.0%)	100 (100%)

Values are median (Q1, Q3) or n (%)

*CT* computed tomography, *CPI* composite physiologic index, *DL*_*CO*_ diffusing capacity of the lungs for carbon monoxide, *FEV*_*1*_ forced expiratory volume in 1 s, *FVC* forced vital capacity

^a^Determined by the investigator according to the 2011 ATS/ERS/JRS/ALAT diagnostic guidelines [15]

^b^Information available for 295 patients

### Associations between MMP/TIMP levels and presence or severity of IPF

**Fig. 1:**
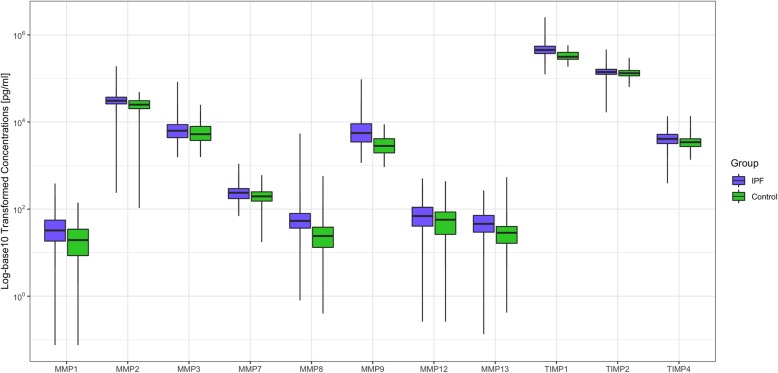
Comparison of MMP or TIMP concentrations in patients with IPF versus the control population

The level of each MMP and TIMP analyzed was significantly higher (corrected *p*-value < 0.05) in patients with IPF versus controls with the exception of TIMP2 (Fig. 1, Table 2, Additional file 2). The highest geometric mean ratios of concentration in patients with IPF versus controls were observed with MMP8 (4.05), MMP1 (2.11) and MMP9 (2.07) (Table 2).

**Table 2:** Associations between MMPs/TIMPs and IPF versus control status, ordered by corrected *p*-value

Protein (pg/mL)	Group	gMean	RatioIPF:control %^a^	Corrected *p*-value^b^
**MMP8**	Control	13.68		
	IPF	55.41	4.05	<.0001
**MMP9**	Control	2801.98		
	IPF	5793.87	2.07	<.0001
**TIMP1**	Control	326,688.87		
	IPF	456,465.47	1.40	<.0001
**MMP1**	Control	11.77		
	IPF	24.88	2.11	<.0001
**TIMP4**	Control	3414.36		
	IPF	4067.08	1.19	0.0004
**MMP7**	Control	196.39		
	IPF	234.88	1.20	0.0008
**MMP2**	Control	22,709.02		
	IPF	29,111.36	1.28	0.0024
**MMP13**	Control	25.20		
	IPF	37.55	1.49	0.0024
**MMP12**	Control	41.85		
	IPF	58.16	1.39	0.0097
**MMP3**	Control	5393.62		
	IPF	6370.79	1.18	0.0164
**TIMP2**	Control	134,417.53		
	IPF	141,289.58	1.05	0.1029

*gMean* geometric mean

^a^Represents the ratio of the geometric mean concentration for each protein in patients with IPF relative to controls. Model includes IPF status (yes/no) as factor

^b^*p*-values were corrected for multiple comparisons using the Benjamini-Hochberg method to control the false discovery rate at 5%

The median (Q1, Q3) concentrations of each MMP/TIMP by tertile of FVC % predicted, DL_CO_ % predicted, and CPI in the IPF cohort are described in Additional file 3. In linear regression analyses, there were no statistically significant associations between levels of MMPs or TIMPs and FVC % predicted. However, higher levels of MMPs 7, 8, 12, and 13 and TIMP4 were significantly associated with lower DL_CO_ % predicted and higher (worse) CPI score (Table 3). Additionally, higher levels of MMP9 were significantly associated with higher CPI (Table 3). MMP7 had the largest estimated effect size in both the DL_CO_ and CPI analyses, followed by TIMP4 (DL_CO_ and CPI) and MMP13 (DL_CO_). Specifically, the estimated differences in DL_CO_ % predicted per 10-fold increase in circulating MMP7, TIMP4, and MMP13 were − 13.41, − 11.80%, and − 6.37%, respectively. For CPI, the estimated difference per 10-fold increase in circulating MMP7 was 11.01 points, while for TIMP4 it was 7.94 points (Table 3). Adjusting these regression analyses for anti-fibrotic treatment did not influence the significance nor the effect estimates of the association between these proteins and disease severity (Additional file 4).

**Table 3:** Association between MMPs/TIMPs and clinical measures of IPF severity

Protein	Association with FVC % predicted			Association with DL_**CO**_ % predicted			Association with CPI		
	Estimated effect (β)^**a**^	Estimated effect^**b**^	Corrected ***p***-value^**c**^	Estimated effect (β)^**a**^	Estimated effect^**b**^	Corrected ***p***-value^**c**^	Estimated effect (β)^**a**^	Estimated effect^**b**^	Corrected ***p***-value^**c**^
**MMP1**	−0.72	−0.0747	0.7547	−1.82	−0.1717	0.2488	1.28	0.1325	0.3279
**MMP2**	4.93	0.0391	0.2859	−1.27	−0.0284	0.7314	0.03	0.0006	0.9886
**MMP3**	2.35	0.0729	0.7547	−3.94	−0.1193	0.2700	2.26	0.0430	0.4260
**MMP7**	−10.22	−0.4649	0.1616	***−13.41***	***−1.0998***	***0.0074***	***11.01***	***0.8748***	***0.0056***
**MMP8**	−6.34	−0.2639	0.1616	***−5.90***	***−0.6893***	***0.0409***	***5.47***	***0.5862***	***0.0151***
**MMP9**	−7.96	−1.2797	0.1518	−3.87	−0.6113	0.2488	***5.34***	***1.0360***	***0.0286***
**MMP12**	−3.90	−0.4665	0.1871	***−5.54***	***−0.5672***	***0.0074***	***4.37***	***0.4930***	***0.0073***
**MMP13**	−2.98	−0.2180	0.2503	***−6.37***	***−0.8935***	***0.0005***	***4.70***	***0.6179***	***0.0016***
**TIMP1**	−1.06	−0.0007	0.8715	0.57	0.0195	0.9173	1.15	0.0242	0.8708
**TIMP2**	−3.36	−0.0026	0.7547	−6.75	−0.0237	0.4094	5.49	0.0379	0.4260
**TIMP4**	−2.66	−0.0059	0.7547	***−11.80***	***−0.9541***	***0.0261***	***7.94***	***0.6367***	***0.0497***

^a^Estimated difference in disease severity measure per 10-fold increase in protein concentration, as determined by the linear regression model

^b^The estimated linear regression coefficients (B) and confidence intervals were used to calculate the estimated difference in the disease severity measure going from the median of tertile 1 to the median of tertile 3 in MMP or TIMP concentration

^c^*p*-value determined by linear regression corrected for multiplicity using the Benjamini-Hochberg method to control the false discovery rate at 5%

Given that prior reports suggest that circulating MMP or TIMP expression can be altered in smoking-related respiratory diseases such as chronic obstructive pulmonary disease and emphysema [21, 22], and a substantial proportion of the patients with IPF and controls in our study were former smokers, we investigated whether there were differences in MMP or TIMP expression by current/past versus never smoking status. No significant differences were observed in either the IPF or control populations (Additional file 5). We also compared MMP and TIMP concentrations between subjects with vs. without clinically significant emphysema on CT scan. These analyses demonstrated that circulating MMP2 was significantly higher among patients with clinically significant radiographic emphysema (Additional file 6). None of the other measured MMPs or TIMPS were statistically different between these two groups. Additionally, among the IPF cohort, none of the MMP/TIMPs analyzed significantly associated with FEV_1_% predicted.

### Sets of MMPs/TIMPs that best discriminate IPF

**Fig. 2:**
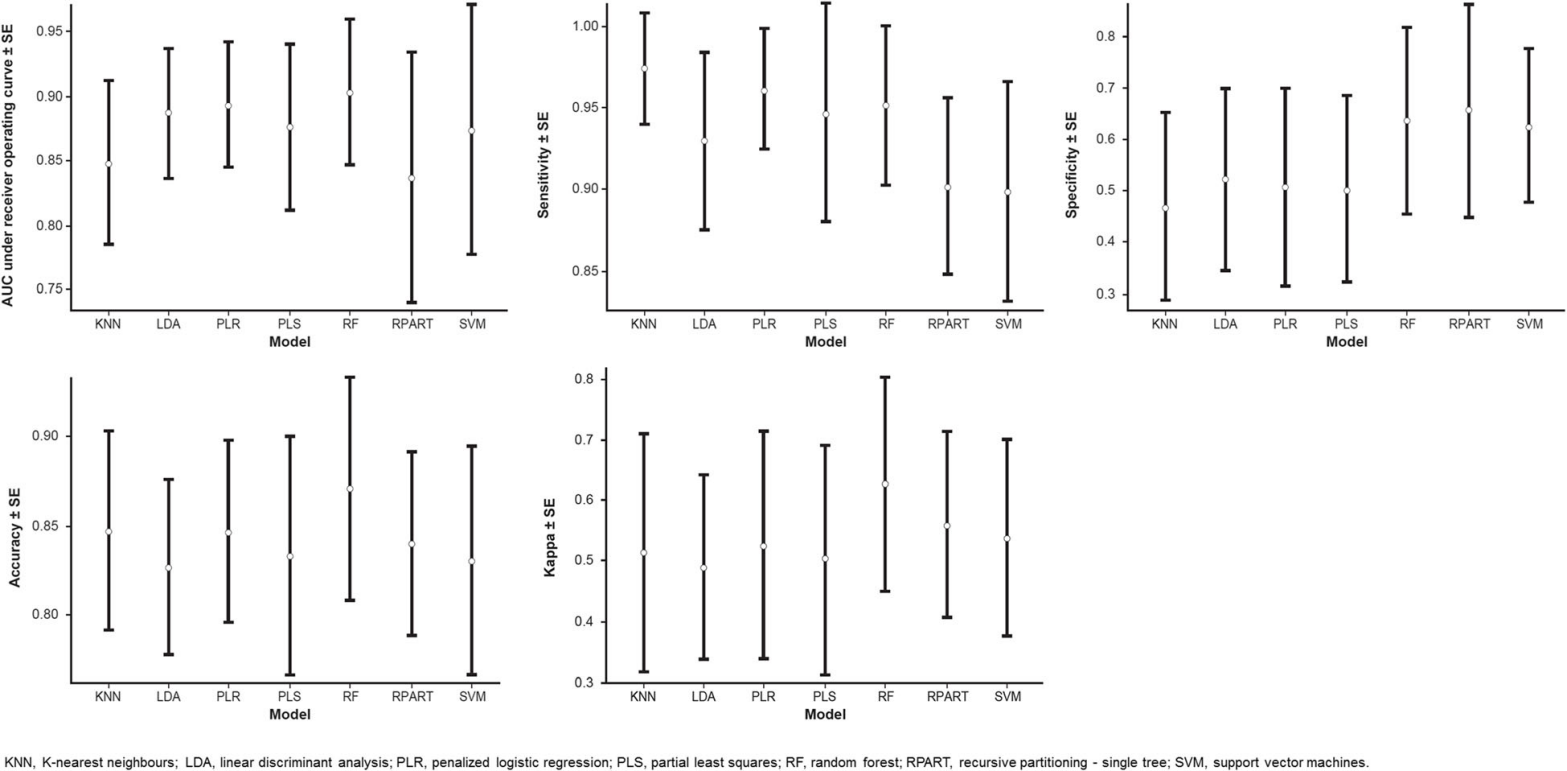
Comparison of operating characteristic of linear and non-linear models to classify IPF versus control status in the training set

**Fig. 3:**
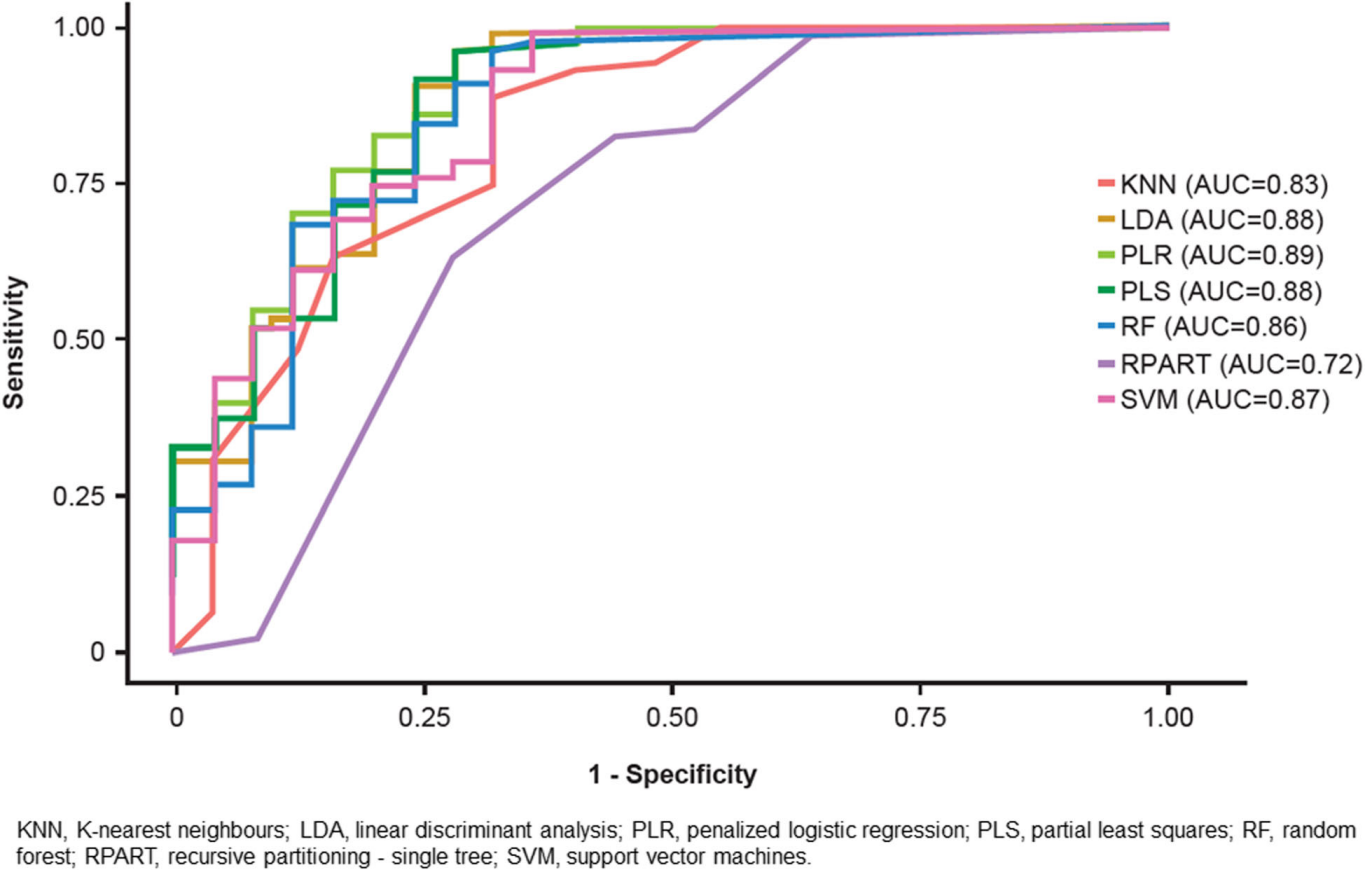
Receiver operating curves for the test data fit across linear and non-linear multivariable models

**Fig. 4:**
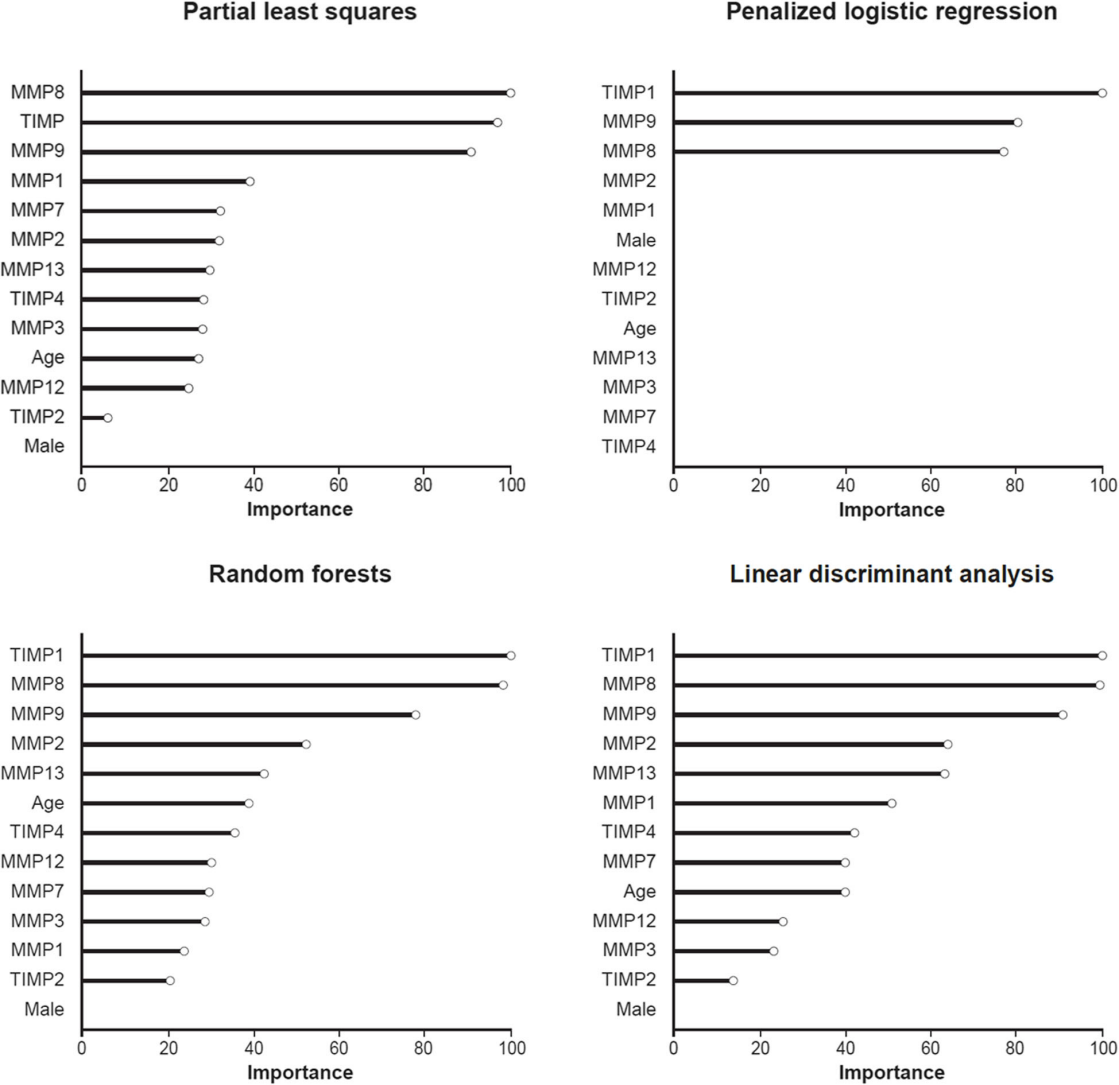
Variable importance of proteins in the three linear multivariable models and the best performing non-linear multivariable model

**Fig. 5:**
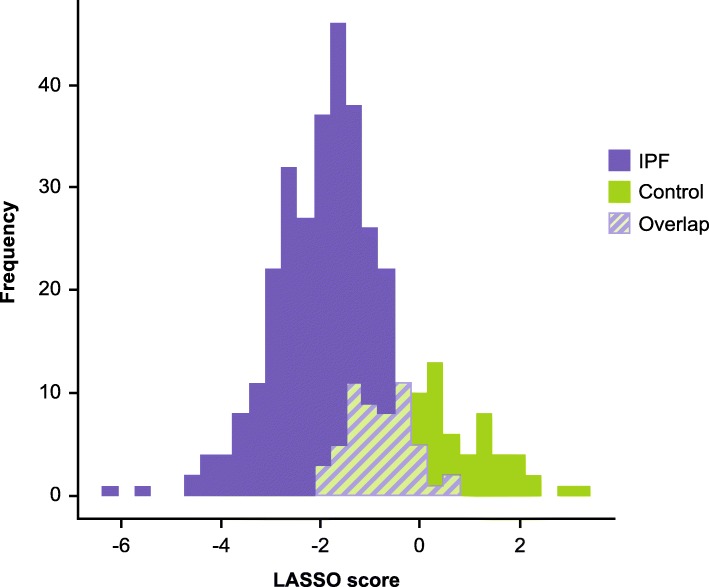
Histogram of the penalized logistic regression model scores for each subject in the IPF and control cohorts

As nearly all of the MMPs and TIMPs measured were elevated in patients with IPF, we sought to understand whether sets of these proteins differentiated patients with IPF from controls better than any single protein. There was no collinearity for the MMPs and TIMPs, thus a multivariable approach may more appropriately account for synergistic or antagonistic relationships that may exist between these molecules. We found that in general, linear multivariable models (penalized logistic regression, partial least squares, and linear discriminant analysis) had similar or better operating characteristics compared with more complex non-linear methods in the training set, with most models obtaining classification accuracies between 80 and 90% over all iterations of the cross-validation procedure (Fig. 2). In the training set, penalized logistic regression was the best performing linear model with an AUC of 0.89 (SD 0.04), while random forests was the best performing non-linear model (AUC 0.90 [SD 0.05]) (Fig. 2). In the test set, based on the AUC, the penalized logistic regression model also performed the best, with a classification accuracy of 0.89 (Fig. 3, Table 4). Variable importance measures were determined for the three linear models and the best performing nonlinear model (random forests). TIMP1, MMP9 and MMP8 were the top proteins of importance across the models (Fig. 4). Next, the penalized logistic regression model was refit to all the data (test and training sets). A histogram of the regression scores for each subject, calculated using the model coefficients for MMP8 (− 0.55), MMP9 (− 0.65) and TIMP1 (− 0.64) and their respective concentrations, is plotted in Fig. 5, with lower scores associating with IPF.

**Table 4:** Operating characteristic of linear and non-linear models to classify IPF versus control status in the test set

Model	AUC	Sensitivity	Specificity	Accuracy	Kappa
PLS	0.88	0.99	0.60	0.89	0.67
PLR	0.89	0.99	0.60	0.89	0.67
LDA	0.88	0.99	0.64	0.90	0.70
SVM	0.87	0.93	0.64	0.86	0.61
KNN	0.83	0.95	0.52	0.84	0.52
RPART	0.72	0.84	0.48	0.75	0.32
RF	0.87	0.96	0.64	0.88	0.65

*AUC* area under the curve, *KNN* K-nearest neighbors, *LDA* linear discriminant analysis, *PLR* penalized logistic regression, *PLS* partial least squares, *RF* random forests, *RPART* recursive partitioning; SVM, support vector machines

### Prediction of IPF disease severity using MMP and TIMP measurements

Multivariable models were fit to understand whether sets of MMPs/TIMPs predicted measures of disease severity in this IPF cohort. The maximum R-squared value achieved for predicting any disease severity measure was 0.16 in the training set (Additional file 7). R-squared values were lower in the test set (Additional file 8). These results indicate that no enhancement in discrimination of disease severity was achieved when considering sets of MMPs/TIMPs.

## Discussion

We present the first study to quantify expression of a broad array of circulating MMPs and TIMPs in a multicenter cohort of well-characterized patients with IPF and in controls without known lung disease. This study not only provides insights into how single MMPs/TIMPs relate to IPF status and severity, but also considers the influence of combinations of these proteins. Our results demonstrate that circulating MMPs and TIMPs are generally elevated among patients with IPF, consistent with altered extracellular matrix remodeling. The combination of MMPs 8 and 9 and TIMP1 demonstrated good performance characteristics in differentiating patients with IPF from controls with a similar distribution of age, sex, and smoking status. Moreover, several MMPs, in addition to TIMP4, strongly associated with CPI. The association between MMPs/TIMPs and CPI appeared to be driven mostly by DL_CO_ % predicted, as no MMP/TIMP significantly associated with FVC % predicted.

In our analyses, circulating levels of MMPs 7, 8, 12, and 13 associated with both DL_CO_ and CPI, while MMP9 associated with CPI only. Though we found MMP2 concentrations to be higher among IPF patients with clinically significant radiographic emphysema, we did not find a significant association between MMP2 and DL_CO_. MMP7 had the largest estimated effect size in our disease severity analyses. Prior work has shown that MMP7 is elevated in patients with IPF [11, 12, 23–25] and is negatively correlated with DL_CO_ [11, 24]. Previous studies have also suggested that circulating MMP7 concentrations increase as FVC % predicted values decline [12] and that elevated MMP7 concentrations may identify patients with IPF with a worse prognosis [13, 23]. However, a recent study found that while MMP7 was elevated in patients with IPF compared with controls, there was no difference in baseline concentrations of MMP7 between patients whose disease progressed or did not progress over a 52-week follow-up period [25].

In addition to supporting the value of MMP7 as a marker of IPF severity, our work identified other MMPs associated with disease severity measures. Previous studies have shown that circulating levels of MMP8 are increased in patients with IPF [9, 11], although they did not correlate with disease severity measures, including DL_CO_, in a cohort of 74 patients [9]. Increased circulating levels of MMP3 and MMP9 in patients with IPF have also been reported, although their association with pulmonary function was not investigated [11]. We found that circulating concentrations of MMPs 12 and 13 were increased in patients with IPF and associated with DL_CO_ and CPI. Studies in murine models of pulmonary fibrosis have yielded inconsistent results regarding the roles of these MMPs after exposure to bleomycin, radiation, or other insults [26–31]. Few clinical data exist regarding circulating levels of these MMPs, but MMP13 has been shown to be overexpressed in the lungs of patients with IPF [30]. Among patients with systemic sclerosis, MMP12 concentrations were increased in patients who had interstitial lung disease compared with those who did not, and correlated with the degree of pulmonary restriction [32]. Together, these observations support further investigation into the potential value of MMPs 12 and 13 to predict clinically relevant outcomes among patients with IPF.

In our IPF cohort, we observed higher levels of TIMP4 to be associated with lower DL_CO_ and higher (worse) CPI. This finding is of interest, as TIMP4 differs from other TIMPs in that at homeostasis, its expression is restricted to cardiovascular structures [33]. Moreover, studies in individuals with pulmonary hypertension have indicated that TIMP4 levels correlate with hemodynamic parameters [34]. It is unknown whether the association we observed between DL_CO_ or CPI and peripheral blood TIMP4 expression reflects pulmonary vascular remodeling and development of pulmonary hypertension, accumulating lung fibrosis, or the effects of angiogenesis supporting pulmonary hypertension and fibroplasia.

Consistent with our results, a prior study implicated MMP8 as a differentiating protein in a 5-protein classifier (MMP1, MMP7, MMP8, IGFBP-1, TNFRS1A) of IPF status, although the classification model did not select MMP9 and did not consider TIMP1 [11]. Our results suggest that the combination of MMP8, MMP9 and TIMP1 provides good discriminatory ability in classifying individuals as having or not having IPF, with TIMP1 being the most important distinguishing variable in two of the best performing models. Collectively, these studies support the role of evaluating multiple MMP/TIMP sets to aid in determining the presence and severity of IPF. Additionally, such data may provide useful guidance for future investigations, such as those focused on MMP/TIMPs that discriminate IPF from other fibrosing lung diseases or those examining patients with early interstitial lung abnormalities of uncertain clinical significance.

When our data on circulating MMP or TIMP levels in patients with IPF are considered in the context of data from experimental models of fibrosis, a clearer view emerges of the potential mechanism(s) by which altered MMP/TIMP expression contributes to lung fibrosis. Both MMP3 and MMP7 appear critical to the development of experimental lung fibrosis, with rodents genetically deficient in MMP3 or MMP7 demonstrating a reduction in pulmonary fibrosis after bleomycin challenge [35–37]. MMP7 can promote pulmonary neutrophil recruitment and chemokine dependent angiogenesis, two important processes in the development of lung fibroplasia [37–42]. The pro-fibrotic nature of MMPs 3 and MMP7 is supported by our observation that higher levels of these MMPs are associated with higher CPI, i.e. more severe disease, among patients with IPF. MMP12 has been found to be pro-fibrotic during bleomycin-induced lung fibrosis [27, 43–45], in line with our findings that elevated levels of MMP12 are associated with worse CPI.

In contrast to these pro-fibrotic MMPs, animal models suggest that augmented levels of MMP1 decrease fibroplasia in liver, muscle and heart [46–48], while MMP2 decreased type I collagen production in experimental liver fibrosis [49]. This could imply that the elevations in circulating MMP1 and MMP2 expression we observed in patients with IPF are a failed attempt to control lung fibrosis. MMP13 cleaves and so reduces the activity of CCL2 and CXCL12 [29, 50]. This suggests that augmented levels of MMP13 may be anti-fibrotic by decreasing the recruitment of CCR2-expressing “pro-fibrotic” macrophages [51] and CCR2- and CXCR4-expressing fibrocytes [52, 53]. TIMP1−/− mice have not been shown to develop reduced lung fibrosis in response to bleomycin [31, 54]; however, our finding of augmented levels of human TIMP1 suggests an unsuccessful attempt at regulating fibrosis in IPF. Collectively, our human data, in conjunction with data from animal models, insinuate that pro-fibrotic mediators (MMP3, MMP7, and particularly MMP12) overwhelm any anti-fibrotic effects mediated by MMP1, MMP2, MMP13, and TIMP1, leading to increased extracellular matrix deposition and impairments in pulmonary function and gas exchange.

While our study has several strengths, including the multicenter nature of the IPF cohort, we acknowledge that it has inherent limitations. First, although we characterized a broad array of MMPs, we did not include all described MMPs nor TIMP3. Additionally, while our MMP assay provides precise quantification of circulating MMP concentrations, MMP activity and organ specificity cannot be inferred. Second, as pulmonary function data were not available for the majority of subjects prior to the date of enrolment, we cannot ascertain whether patients were experiencing significant disease progression or were relatively stable at the time of sampling. Finally, although this work contributes important new information regarding circulating MMP and TIMP expression in patients with IPF, our study was not designed to understand whether the observed changes are specific to IPF as compared with non-IPF fibrosing lung diseases. As the IPF-PRO Registry has recently expanded to include patients with non-IPF interstitial lung diseases in the ILD-PRO Registry, we anticipate that future studies will address the specificity of candidate biomarkers for IPF as compared with other fibrosing lung diseases.

## Conclusion

The results of this study further delineate the potential value of selected MMPs or TIMPs as disease-related biomarkers in patients with IPF. Further validation will be necessary, as will extension of these analyses to examine associations between MMP/TIMP expression and clinical outcomes. Rich longitudinal data collected in the IPF-PRO Registry, including serial pulmonary function measures, hospitalization data, and information on vital status will support these analyses and further the goal of improving the diagnosis and management of patients with IPF.

## Supplementary information

**Additional file 1:** Tertile cutpoints for disease severity metrics.

**Additional file 2:** MMP and TIMP concentrations (pg/mL) in patients with IPF versus the control population.

**Additional file 3:** Median (first quartile, third quartile) concentration for each MMP/TIMP stratified by tertile of FVC % predicted, DLCO % predicted, and CPI.

**Additional file 4:** Association of MMPs and TIMPs with measures of IPF severity, adjusted for anti-fibrotic treatment.

**Additional file 5:** Mean (standard deviation) log10 MMP and TIMP concentrations in the IPF and control populations as stratified by smoking status at enrollment (current/past smoker vs. never smoker).

**Additional file 6:** Mean (standard deviation) log10 MMP and TIMP concentrations in patients with IPF with or without clinically significant emphysema on CT scan as assessed by the enrolling physician.

**Additional file 7:** Model performance in the training (cross-validation) set for baseline FVC % predicted, DLCO % predicted and CPI.

**Additional file 8:** Model performance in the test set for baseline FVC % predicted, DLco % predicted and CPI.

## Data Availability

A summary of data generated or analyzed during this study are included in this published article or its supplementary information files.
