# Supplementary material for: Circulating matrix metalloproteinases and tissue metalloproteinase inhibitors in patients with idiopathic pulmonary fibrosis in the multicenter IPF-PRO Registry cohort
Source: BMC Pulm Med. 2020 Mar 14;20:64. doi: 10.1186/s12890-020-1103-4 (PMC7071646; doi:10.1186/s12890-020-1103-4)
Supplement: Supplementary file 1 — Additional file 1: Tertile cutpoints for disease severity metrics. [file 12890_2020_1103_MOESM1_ESM.pdf]

Additional file 1. Tertile cutpoints for disease severity metrics.

Metric	Lower Cutpoint	Upper Cutpoint
CPI	49.40	58.39
FVC % predicted	63.70	76.76
DL_{CO} % predicted	35.20	46.95

CPI, composite physiologic index; DL_{CO}, diffusing capacity of the lungs for carbon monoxide; FVC, forced vital capacity.
