# Supplementary material for: Circulating matrix metalloproteinases and tissue metalloproteinase inhibitors in patients with idiopathic pulmonary fibrosis in the multicenter IPF-PRO Registry cohort
Source: BMC Pulm Med. 2020 Mar 14;20:64. doi: 10.1186/s12890-020-1103-4 (PMC7071646; doi:10.1186/s12890-020-1103-4)
Supplement: Supplementary file 2 — Additional file 2: MMP and TIMP concentrations (pg/mL) in patients with IPF versus the control population. [file 12890_2020_1103_MOESM2_ESM.pdf]

Additional file 2. MMP and TIMP concentrations (pg/mL) in patients with IPF versus the control population.

Protein	Population	N	Min	25th percentile	Median	75th percentile	Max	Mean	Standard deviation
**MMP1**	IPF	300	0.08	17.91	32.37	55.81	387.39	43.54	44.10
	Control	100	0.08	8.34	19.42	35.27	139.64	25.33	26.48
**MMP2**	IPF	300	236.90	26067.67	30926.62	37076.40	191694.82	32555.21	14415.48
	Control	100	106.21	20288.37	25064.32	30903.38	48623.54	25808.48	8409.41
**MMP3**	IPF	300	1552.25	4379.23	6310.00	8730.98	83631.97	7994.32	8096.36
	Control	100	1568.54	3776.16	5252.57	7909.93	24830.73	6185.21	3525.26
**MMP7**	IPF	300	69.05	174.53	237.63	295.73	1100.09	259.06	128.78
	Control	100	17.48	152.72	195.96	249.38	602.93	215.07	94.25
**MMP8**	IPF	300	0.80	36.56	53.61	79.40	5417.52	88.26	316.60
	Control	100	0.40	13.21	24.10	38.42	575.63	34.91	62.94
**MMP9**	IPF	300	1158.97	3481.40	5586.75	9086.73	95513.71	7507.49	7742.25
	Control	100	932.56	1951.24	2827.69	4136.99	8816.96	3203.14	1735.13
**MMP12**	IPF	300	0.26	40.58	69.22	110.46	505.53	82.14	62.65
	Control	100	0.26	26.25	57.01	86.79	436.81	65.90	62.21
**MMP13**	IPF	300	0.14	29.60	45.83	71.47	267.87	53.79	37.84
	Control	100	0.42	16.42	28.60	40.03	538.16	36.27	55.70

**TIMP1**	IPF	300	124656.12	375017.66	451095.35	550093.92	2542100.00	485624.83	204982.95
	Control	100	188139.04	276633.79	315033.43	397911.11	575475.47	337874.12	88977.95
**TIMP2**	IPF	300	16688.30	124691.26	140383.81	162050.16	461355.33	146066.92	39837.75
	Control	100	63550.51	114663.24	132096.89	152272.27	298072.74	139149.69	39189.23
**TIMP4**	IPF	300	391.49	3203.04	4100.75	5191.03	13549.28	4392.84	1735.13
	Control	100	1359.12	2730.31	3435.03	4115.11	13674.65	3674.56	1648.70
