# Supplementary material for: Circulating matrix metalloproteinases and tissue metalloproteinase inhibitors in patients with idiopathic pulmonary fibrosis in the multicenter IPF-PRO Registry cohort
Source: BMC Pulm Med. 2020 Mar 14;20:64. doi: 10.1186/s12890-020-1103-4 (PMC7071646; doi:10.1186/s12890-020-1103-4)
Supplement: Supplementary file 3 — Additional file 3: Median (first quartile, third quartile) concentration for each MMP/TIMP stratified by tertile of FVC % predicted, DLCO % predicted, and CPI. [file 12890_2020_1103_MOESM3_ESM.pdf]

Additional file 3. Median (first quartile, third quartile) concentration for each MMP/TIMP stratified by tertile of FVC % predicted, DL_{CO} % predicted, and CPI.

Protein (pg/mL)	FVC % predicted			DL_{CO} % predicted			CPI		
	Tertile 1	Tertile 2	Tertile 3	Tertile 1	Tertile 2	Tertile 3	Tertile 1	Tertile 2	Tertile 3
**MMP1**	39.07 (23.20-62.18)	26.14 (12.75-51.78)	30.79 (15.07-54.66)	38.42 (23.11-67.49)	29.48 (15.71-50.21)	30.92 (11.89-51.66)	30.78 (12.76-49.74)	30.16 (15.08-52.00)	39.07 (23.20-67.49)
**MMP2**	30344.02 (26053.51-37069.72	31586.16 (27418.90-37921.57	29794.79 (25257.21-36554.29	30923.93 (26624.82-36865.93)	31459.37 (26694.17-38664.93)	29374.21 (24883.76-36306.47)	29634.31 (23732.83-36377.50)	31568.39 (26733.15-37039.57)	30923.93 (26834.08-37845.31)
**MMP3**	6531.08 (4651.99-8300.57	6332.08 (3938.06-9056.61)	6080.23 (4380.26-9176.99)	6476.34 (4483.32-9806.68)	6349.97 (4114.35-8837.38)	6040.09 (4400.39-8290.76)	6198.11 (4414.60-8531.70)	6272.61 (4024.62-7762.02)	6476.34 (4548.86-10282.81)
**MMP7**	247.85 (203.14-325.19)	224.20 (165.39-328.87)	223.20 (173.68-277.17)	258.21 (212.56-332.91)	226.76 (169.75-286.00)	213.78 (165.74-268.95)	215.05 (160.51-266.38)	229.54 (170.35-295.73)	258.21 (211.81-335.45)
**MMP8**	60.61 (39.79-95.14)	49.24 (31.66-79.04)	55.07 (38.85-68.42)	62.92 (44.39-113.75)	51.20 (36.04-67.77)	48.07 (34.53-68.69)	49.87 (34.92-62.58)	48.42 (35.62-72.89)	63.81 (42.64-113.75)
**MMP9**	7076.01 (3656.02-10058.80)	5491.60 (3337.91-8702.68)	4887.53 (3446.82-8150.05)	6919.93 (3825.67-10324.59)	5723.32 (3436.55-8855.98)	4809.11 (3238.35-7539.73)	4812.21 (3282.68-7666.10)	5211.92 (3157.26-8190.94)	7523.44 (4517.84-12327.98)
**MMP12**	81.78 (49.16-124.95)	51.95 (29.46-105.18)	62.10 (40.96-102.61)	76.44 (45.87-135.70)	64.75 (40.85-100.88)	60.38 (33.93-96.55)	60.08 (35.76-96.46)	64.22 (38.09-105.61)	77.91 (47.12-131.95)

**MMP13**	55.09 (37.77-84.82)	39.99 (19.17-64.29)	46.54 (30.45-65.78)	54.85 (34.23-85.81)	44.27 (30.29-64.61)	39.72 (23.68-68.25)	40.93 (26.59-67.70)	42.58 (27.55-61.13)	55.38 (37.30-87.55)
**TIMP1**	453511.85 (387387.95-535856.35)	446308.00 (357390.30-562568.85)	452813.85 (367331.35-525346.05)	435606.25 (366875.90-535856.35)	446919.50 (357212.90-570017.90)	471149.40 (386315.60-534450.30)	466500.36 (389536.37-525346.10)	443417.57 (353860.36-568303.27)	444377.46 (374224.59-548099.39)
**TIMP2**	139618.80 (125239.40-155392.85)	143464.00 (123742.05-167573.20)	139869.70 (125058.45-159254.80)	141497.90 (125518.70-160997.40)	138877.30 (125066.70-167393.50)	140357.90 (124612.40-158020.90)	139410.98 (124100.43-156414.57)	139842.16 (124458.15-163596.04)	141645.31 (126452.64-162541.19)
**TIMP4**	4235.14 (3355.91-5372.85)	3948.40 (2960.04-5132.03)	4256.79 (3172.93-5058.68)	4579.19 (3282.03-5563.62)	3972.20 (3157.18-5227.46)	3801.53 (3161.46-4825.90)	3920.26 (3175.07-4982.71)	3868.34 (2960.62-4907.14)	4714.83 (3406.45-5824.77)

CPI, composite physiologic index; DL_{CO}, diffusing capacity of the lungs for carbon monoxide; FVC, forced vital capacity.
