# Supplementary material for: Circulating matrix metalloproteinases and tissue metalloproteinase inhibitors in patients with idiopathic pulmonary fibrosis in the multicenter IPF-PRO Registry cohort
Source: BMC Pulm Med. 2020 Mar 14;20:64. doi: 10.1186/s12890-020-1103-4 (PMC7071646; doi:10.1186/s12890-020-1103-4)
Supplement: Supplementary file 4 — Additional file 4: Association of MMPs and TIMPs with measures of IPF severity, adjusted for anti-fibrotic treatment. [file 12890_2020_1103_MOESM4_ESM.pdf]

Additional file 4. Association of MMPs and TIMPs with measures of IPF severity, adjusted for anti-fibrotic treatment.

	**Association with FVC % predicted**		**Association with DL_{CO} % predicted**		**Association with CPI**	
**Protein**	**Estimated effect* (β)**	**Corrected *p*-value†**	**Estimated effect* (β)**	**Corrected *p*-value†**	**Estimated effect* (β)**	**Corrected *p*-value†**
**MMP1**	-0.69	0.7391	-1.79	0.2615	1.27	0.3376
**MMP2**	4.86	0.3012	-1.38	0.7044	0.08	0.9725
**MMP3**	2.34	0.7391	-4.01	0.2615	2.26	0.4278
**MMP7**	-10.49	0.1465	***-13.63***	***0.0080***	***11.20***	***0.0049***
**MMP8**	-6.49	0.1465	***-6.06***	***0.0359***	***5.57***	***0.0140***
**MMP9**	-8.14	0.1359	-3.97	0.2615	***5.44***	***0.0260***
**MMP12**	-3.91	0.1942	***-5.53***	***0.0084***	***4.42***	***0.0073***
**MMP13**	-2.97	0.2547	***-6.36***	***0.0006***	***4.71***	***0.0017***
**TIMP1**	-1.48	0.8237	0.22	0.9684	1.37	0.8309
**TIMP2**	-3.54	0.7391	-6.82	0.4064	5.61	0.4278
**TIMP4**	-2.83	0.7391	***-11.90***	***0.0257***	***8.10***	***0.0460***

*Estimated effect (β) is the estimated difference in disease severity measure per 10-fold increase in protein concentration, as determined by linear regression.

†*p*-value determined by linear regression corrected for multiplicity using the Benjamini-Hochberg method to control the false discovery rate at 5%.

CPI, composite physiologic index; DL_{CO}, diffusing capacity of the lungs for carbon monoxide; FVC, forced vital capacity.
