# Supplementary material for: Circulating matrix metalloproteinases and tissue metalloproteinase inhibitors in patients with idiopathic pulmonary fibrosis in the multicenter IPF-PRO Registry cohort
Source: BMC Pulm Med. 2020 Mar 14;20:64. doi: 10.1186/s12890-020-1103-4 (PMC7071646; doi:10.1186/s12890-020-1103-4)
Supplement: Supplementary file 5 — Additional file 5: Mean (standard deviation) log10 MMP and TIMP concentrations in the IPF and control populations as stratified by smoking status at enrollment (current/past smoker vs. never smoker). [file 12890_2020_1103_MOESM5_ESM.pdf]

**Additional file 5.** Mean (standard deviation) log10 MMP and TIMP concentrations in the IPF and control populations stratified by smoking status at enrollment (current/past smoker vs. never smoker).

| Protein (pg/mL)* | Control | | IPF | |
|---|---|---|---|---|
| | Current/Past Smoker (N=68) | Never Smoker (N=32) | Current/Past Smoker (N=204) | Never Smoker (N=96) |
| **MMP1** | 1.07 (0.82) | 1.08 (0.64) | 1.38 (0.67) | 1.42 (0.53) |
| **MMP2** | 4.36 (0.32) | 4.34 (0.38) | 4.45 (0.32) | 4.49 (0.12) |
| **MMP3** | 3.74 (0.22) | 3.72 (0.25) | 3.82 (0.27) | 3.77 (0.25) |
| **MMP7** | 2.32 (0.17) | 2.24 (0.24) | 2.38 (0.18) | 2.35 (0.20) |
| **MMP8** | 1.16 (0.76) | 1.08 (0.85) | 1.75 (0.35) | 1.73 (0.20) |
| **MMP9** | 3.45 (0.23) | 3.44 (0.23) | 3.77 (0.30) | 3.75 (0.29) |
| **MMP12** | 1.63 (0.49) | 1.61 (0.57) | 1.75 (0.47) | 1.80 (0.40) |
| **MMP13** | 1.38 (0.38) | 1.45 (0.33) | 1.58 (0.49) | 1.56 (0.55) |
| **TIMP1** | 5.51 (0.11) | 5.51 (0.12) | 5.66 (0.15) | 5.66 (0.13) |
| **TIMP2** | 5.13 (0.11) | 5.12 (0.12) | 5.14 (0.12) | 5.16 (0.10) |
| **TIMP4** | 3.53 (0.17) | 3.55 (0.15) | 3.60 (0.19) | 3.64 (0.14) |

*Results of t-tests for differences in MMP/TIMP concentrations between current/past smokers vs never smokers within the control and IPF populations, respectively, indicated no statistically significant differences.
