# Supplementary material for: Circulating matrix metalloproteinases and tissue metalloproteinase inhibitors in patients with idiopathic pulmonary fibrosis in the multicenter IPF-PRO Registry cohort
Source: BMC Pulm Med. 2020 Mar 14;20:64. doi: 10.1186/s12890-020-1103-4 (PMC7071646; doi:10.1186/s12890-020-1103-4)
Supplement: Supplementary file 6 — Additional file 6: Mean (standard deviation) log10 MMP and TIMP concentrations in patients with IPF with or without clinically significant emphysema on CT scan as assessed by the enrolling physician. [file 12890_2020_1103_MOESM6_ESM.pdf]

Additional file 6. Mean (standard deviation) log10 MMP and TIMP concentrations in patients with IPF with or without clinically significant emphysema on CT scan as assessed by the enrolling physician.

**Protein (pg/mL)**	**IPF**		
	**Clinically Significant Emphysema Present on CT scan (N=31)**	**Clinically Significant Emphysema Absent on CT scan (N=269)**	**P-value***
**MMP1**	1.39 (0.76)	1.40 (0.61)	0.9388
**MMP2**	4.56 (0.21)	4.45 (0.28)	0.0478
**MMP3**	3.91 (0.34)	3.79 (0.25)	0.0706
**MMP7**	2.42 (0.15)	2.36 (0.19)	0.0991
**MMP8**	1.80 (0.37)	1.74 (0.32)	0.2952
**MMP9**	3.82 (0.35)	3.76 (0.29)	0.2811
**MMP12**	1.71 (0.53)	1.77 (0.44)	0.5118
**MMP13**	1.45 (0.77)	1.59 (0.47)	0.1596
**TIMP1**	5.68 (0.15)	5.66 (0.15)	0.3108
**TIMP2**	5.17 (0.09)	5.15 (0.12)	0.2283
**TIMP4**	3.66 (0.18)	3.60 (0.18)	0.1226

*Results of t-tests for differences in MMP/TIMP concentrations between IPF patients with vs. without clinically significant emphysema on CT scan.
