# Supplementary material for: Circulating matrix metalloproteinases and tissue metalloproteinase inhibitors in patients with idiopathic pulmonary fibrosis in the multicenter IPF-PRO Registry cohort
Source: BMC Pulm Med. 2020 Mar 14;20:64. doi: 10.1186/s12890-020-1103-4 (PMC7071646; doi:10.1186/s12890-020-1103-4)
Supplement: Supplementary file 7 — Additional file 7: Model performance in the training (cross-validation) set for baseline FVC % predicted, DLCO % predicted and CPI. [file 12890_2020_1103_MOESM7_ESM.pdf]

Additional file 7. Model performance in the training (cross-validation) set for baseline FVC % predicted, DL_{CO} % predicted and CPI.

	**FVC % predicted**		**DL_{CO} % predicted**		**CPI**	
	**RMSE**	**R^2**	**RMSE**	**R^2**	**RMSE**	**R^2**
**PLS**	11.43	0.16	11.43	0.16	11.11	0.11
**PLR**	16.60	0.07	14.39	0.11	11.16	0.11
**SVM**	16.33	0.07	14.23	0.11	11.03	0.12
**KNN**	16.51	0.05	14.83	0.04	11.23	0.10
**RPART**	16.67	0.07	14.68	0.06	10.94	0.13
**RF**	16.32	0.07	14.02	0.12	10.95	0.12

KNN, K-nearest neighbors; PLR, penalized logistic regression; PLS, partial least squares; RF, random forests; RMSE, root mean squared error; RPART, recursive partitioning; SVM, support vector machines.
