# Supplementary material for: Circulating matrix metalloproteinases and tissue metalloproteinase inhibitors in patients with idiopathic pulmonary fibrosis in the multicenter IPF-PRO Registry cohort
Source: BMC Pulm Med. 2020 Mar 14;20:64. doi: 10.1186/s12890-020-1103-4 (PMC7071646; doi:10.1186/s12890-020-1103-4)
Supplement: Supplementary file 8 — Additional file 8: Model performance in the test set for baseline FVC % predicted, DLco % predicted and CPI. [file 12890_2020_1103_MOESM8_ESM.pdf]

Additional file 8. Model performance in the test set for baseline FVC % predicted, DL_{CO} % predicted and CPI.

	**FVC % predicted**		**DL_{CO} % predicted**		**CPI**	
	**RMSE**	**R^2**	**RMSE**	**R^2**	**RMSE**	**R^2**
**PLS**	26.19	0.02	16.70	<0.01	9.61	<0.01
**PLR**	15.51	0.06	11.74	<0.01	9.63	0.01
**SVM**	16.10	<0.01	10.48	<0.01	8.99	0.04
**KNN**	15.94	<0.01	11.11	0.06	8.93	0.04
**RPART**	16.66	<0.01	10.59	0.06	9.89	<0.01
**RF**	16.40	<0.01	10.72	0.05	9.35	0.01

KNN, K-nearest neighbors; PLR, penalized logistic regression; PLS, partial least squares; RF, random forests; RMSE, root mean squared error; RPART, recursive partitioning; SVM, support vector machines.
